# Repertoire Enhancement with Adoptively Transferred Female Lymphocytes Controls the Growth of Pre-Implanted Murine Prostate Cancer

**DOI:** 10.1371/journal.pone.0035222

**Published:** 2012-04-06

**Authors:** Robert R. Jenq, Michael A. Curran, Gabrielle L. Goldberg, Chen Liu, James P. Allison, Marcel R. M. van den Brink

**Affiliations:** 1 Department of Immunology and Medicine, Memorial Sloan-Kettering Cancer Center, New York, New York, United States of America; 2 Weill Cornell Medical College, New York, New York, United States of America; 3 Howard Hughes Medical Institute, Department of Immunology, Memorial Sloan-Kettering Cancer Center, New York, New York, United States of America; 4 Department of Pathology, Immunology and Laboratory Medicine, University of Florida, College of Medicine, Gainesville, Florida, United States of America; Johns Hopkins University School of Medicine, United States America

## Abstract

**Background:**

In prostate cancer, genes encoding androgen-regulated, Y-chromosome-encoded, and tissue-specific antigens may all be overexpressed. In the adult male host, however, most high affinity T cells targeting these potential tumor rejection antigens will be removed during negative selection. In contrast, the female mature T-cell repertoire should contain abundant precursors capable of recognizing these classes of prostate cancer antigens and mediating effective anti-tumor immune responses.

**Methodology/Principal Findings:**

We find that syngeneic TRAMP-C2 prostatic adenocarcinoma cells are spontaneously rejected in female hosts. Adoptive transfer of naïve female lymphocytes to irradiated male hosts bearing pre-implanted TRAMP-C2 tumor cells slows tumor growth and mediates tumor rejection in some animals. The success of this adoptive transfer was dependent on the transfer of female CD4 T cells and independent of the presence of CD25-expressing regulatory T cells in the transferred lymphocytes. We identify in female CD4 T cells stimulated with TRAMP-C2 a dominant MHC II-restricted response to the Y-chromosome antigen DBY. Furthermore, CD8 T cell responses in female lymphocytes to the immunodominant MHC I-restricted antigen SPAS-1 are markedly increased compared to male mice. Finally, we find no exacerbation of graft-versus-host disease in either syngeneic or minor-antigen mismatched allogeneic lymphocyte adoptive transfer models by using female into male versus male into male cells.

**Conclusions/Significance:**

This study shows that adoptively transferred female lymphocytes, particularly CD4 T cells, can control the outgrowth of pre-implanted prostatic adenocarcinoma cells. This approach does not significantly worsen graft-versus-host responses suggesting it may be viable in the clinic. Further, enhancing the available immune repertoire with female-derived T cells may provide an excellent pool of prostate cancer reactive T cells for further augmentation by combination with either vaccination or immune regulatory blockade strategies.

## Introduction

Despite improvements in detection and treatment, prostate cancer (CaP) remains the second leading cause of cancer death in men in the United States and the second most common cancer in men worldwide. When localized, CaP is often curable through first line therapies such as prostatectomy. Few curative therapies exist, however, for recurrent or metastatic CaP. Recently, sipuleucel-T, a vaccine designed to elicit an immune response against prostatic acid phosphatase, was shown to improve median survival of patients with metastatic castration-resistant CaP [1]. Blockade of the immune regulatory molecule CTLA-4 has also shown clinical promise for prostate cancer [2], [3]. Development of immunotherapeutic interventions which target multiple aspects of the immune system in CaP may offer greater clinical benefit and more durable responses. Development of immunotherapeutic interventions which target multiple antigens in CaP may offer greater clinical benefit and more durable responses.

Prostate cancers likely express a constellation of potential tumor antigens including mutated self-proteins [4], overexpressed androgen-responsive and androgen-regulated proteins [5], [6], and differentially expressed tissue-specific and male Y-linked proteins [7]. T cells developing in males which recognize any of these androgen-related or Y-chromosome antigens with high affinity are likely deleted during negative selection in the thymus. Low affinity T cells which escape central deletion are in turn usually anergized through peripheral tolerance mechanisms upon subsequent antigen encounter. In contrast, the mature female T-cell repertoire should contain an ample frequency of T-cell precursors capable of recognizing all of the above classes of prostate cancer antigens.

Allogeneic hematopoietic stem cell transplantation (allo-HSCT) is one means of altering a patient’s immune repertoire and overcoming immune regulatory mechanisms. Rigorous chemotherapeutic and/or radiation conditioning programs ablate the host’s hematopoietic system, which is then reconstituted with bone marrow or mobilized stem cells from a suitable donor. These donor cells expand in the patient giving rise to a new immune system which can recognize malignant cells in the patient as foreign in a process termed the graft-versus-tumor (GVT) effect [8], [9]. GVT can lead to complete and lasting remissions in patients with cancers that cannot otherwise be cured with chemotherapy or radiation. For this reason, allo-HSCT is widely utilized in the treatment of a variety of hematologic malignancies.

In addition to mediating desirable GVT effects, donor immune cells can also attack normal host tissues resulting in graft-versus-host disease (GVHD). GVHD in both the acute and chronic forms is a major source of morbidity and mortality in recipients of allo-HSCT. Choice of allogeneic donor is known to potentially impact on the risk of developing GVHD; male recipients of female grafts, for example, have an elevated risk of both acute and chronic GVHD, particularly those with female donors who have undergone multiple pregnancies [10]–[12].

Adoptive transfer of autologous *ex vivo* expanded tumor-infiltrating or engineered tumor-specific T cells has shown curative potential for both metastatic melanoma and chronic lymphoid leukemia [13]. These approaches, however, require considerable expertise and often weeks of culturing to expand T cells. By adoptively transferring syngeneic naïve female lymphocytes into male mice pre-implanted with prostate tumors, we sought to test the potential of the female T-cell repertoire to recognize and attack CaP. To improve the expansion of adoptively transferred cells as well as reduce the risk for GVHD and potentially augment GVT effects, we treated recipient mice with nonmyeloablative doses of radiation, given prior reports that a mixed chimeric state can promote GVT responses while minimizing GVHD [14], [15].

## Results

### Female Mice Immunologically Reject TRAMP-C2 Prostatic Adenocarcinoma Cells

In the transgenic adenocarcinoma of mouse prostate (TRAMP) model, tumors that develop are initially androgen-dependent but later become androgen-independent, recapitulating the normal progression of prostate cancer in humans [16]. For our studies, we utilized the TRAMP-C2 cell line derived from these mice. These transplantable CaP cells form tumors when implanted into C57BL6 mice, are partially androgen-sensitive *in vitro*, and express a mutated version of the SPAS-1 protein which is the target of the dominant CD8 T-cell response [17].

**Figure 1:**
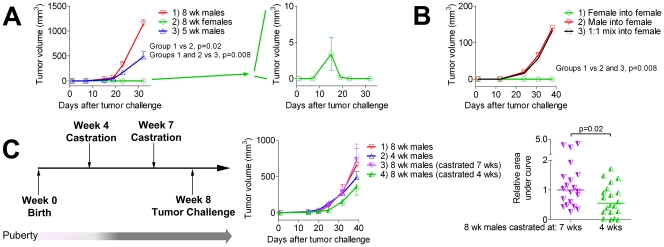
Female mice reject TRAMP-C2 prostate cancers while male mice develop tolerance following puberty. A) TRAMP-C2 tumor cells grow progressively in adult male mice, with a slower kinetic but complete penetrance in pre-pubescent male mice, and grow briefly but then are rejected in adult female mice. n = 5 mice/group, one of 2 experiments with similar results. B) Adult female mice were transplanted with female, male, or a 1∶1 mixture of female and male T-cell depleted bone marrow and 10 weeks after transplantation challenged with TRAMP-C2 tumors. n = 5 mice/group, results of a single experiment. C) Preventing onset of puberty in male mice can mediate delayed growth of TRAMP-C2 tumors. Adult male mice surgically castrated before onset of puberty (4 weeks) show delayed growth of TRAMP-C2, while castration 1 week prior to tumor challenge (7 weeks) had no effect on tumor growth. n = 10 mice/group, tumor growth curve and area under curve normalized to median value for group 3 from each experiment. Combined results of 2 experiments.

We challenged female mice, young male mice, and mature male mice with TRAMP-C2 cells and measured the growth of tumors over time (Figure 1A). Tumors grew robustly in adult male mice, but grew more slowly in young male mice perhaps due to either lower androgen levels or lack of immune tolerance to androgen-regulated genes. In female mice, however, TRAMP-C2 tumors grew initially and then spontaneously regressed. The ability of these tumors to grow initially suggested that their disappearance was due to immunological rejection rather than a dependence on high levels of androgens for sustained growth. To confirm this hypothesis, we repeated the tumor challenge in bone marrow chimeras in which we transplanted female and male T-cell depleted bone marrow into female hosts. TRAMP-C2 tumors were completely rejected in female-into-female chimeras, whereas in male-into-female chimeras TRAMP-C2 tumors grew progressively (Figure 1B). Interestingly, female mice transplanted with a mixture of female and male bone marrow were also unable to mediate tumor rejection. This experiment demonstrates that these prostate cancer cells do not require male androgen levels for sustained growth *in vivo*, and that their rejection in female mice is mediated by the hematolymphoid system. Furthermore, the presence of male hematopoietic cells appears to have a dominant suppressive effect on the ability of female hematopoietic cells to reject TRAMP-C2 tumors, perhaps due to thymic negative selection mediated by expression of male antigens in bone marrow-derived thymic dendritic cells.

The results from the male-into-female chimera mice also implied that the impaired growth of TRAMP-C2 cells in pre-pubescent 5-week old mice was likely due to incomplete immune tolerance to adult-male prostate cancer rather than a lack of sufficient androgen levels. To more directly evaluate for the potential impact of androgen levels as well as other age-induced differences, we challenged adult male mice of the same age (8 weeks) that were surgically castrated either early (at 4 weeks of age prior to onset of puberty), or late (one week prior to tumor challenge). We found that adult male mice that had not undergone puberty due to early castration displayed significantly delayed growth of TRAMP-C2 tumors (Figure 1C). While TRAMP-C2 cells are routinely cultured in the presence of dihydrotestosterone [16], our results demonstrate that TRAMP-C2 can develop progressive tumors *in vivo* in the absence of testes-derived androgens, possibly due to the presence of androgens originating from other endocrine organs such as androstenedione produced by the adrenal glands. Indeed, the TRAMP mouse model, from which TRAMP-C2 originates, is known to produce prostate adenocarcinomas that can progress despite castration [18]. Our data further suggest that while some potential for immune recognition of mature prostate antigens exists in males at an early age, tolerance against these prostate antigens is developed following puberty and leads to accelerated tumor growth.

### Adoptive Transfer of Female Lymphocytes into Male Mice Pre-implanted with TRAMP-C2 Cells Slows Tumor Growth and Occasionally Mediates Tumor Rejection

**Figure 2:**
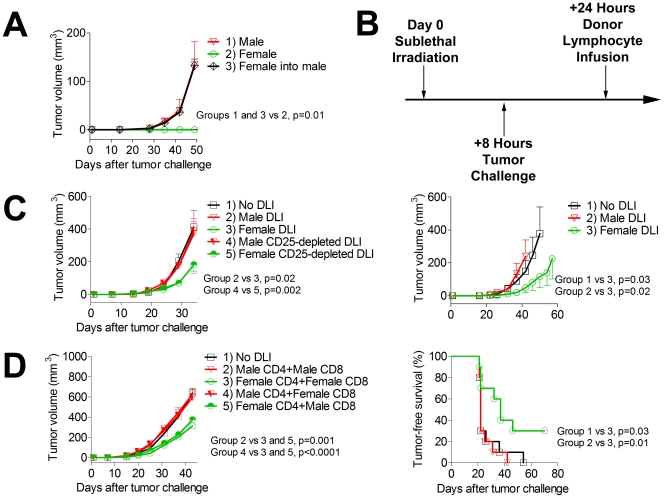
Adoptive transfer of female lymphocytes into male mice challenged with TRAMP-C2 controls tumor growth. A) Adult male mice were myeloablated via irradiation and transplanted with female T-cell depleted bone marrow followed by challenge with TRAMP-C2 tumors 10 weeks after transplantation. n = 5 mice/group, results of a single experiment. B) Adult male mice were sublethally irradiated with 6 Gy and challenged intradermally with 1×10^6^ TRAMP-C2. The following day, mice received a donor lymphocyte infusion (DLI) of 3×10^7^ splenocytes *i.v*. n = 10 mice/group, one of 3 experiments with similar results. C) Adoptively-transferred CD25+ regulatory T cells do not mediate increased tumor growth seen following male DLI. Mice were irradiated and tumor-challenged as in B, and received splenocytes that were CD25-depleted using magnetic beads where indicated. n = 10 mice/group, results of a single experiment. D) Female CD4 T cells mediate delayed growth of TRAMP-C2 tumors. Mice were irradiated and tumor-challenged as in B, and then received 1.5×10^7^ splenocytes that were *in vivo* CD4-depleted, mixed with 1.5×10^7^ splenocytes that were CD8-depleted, of either male or female origin. n = 10 mice/group, one of 2 experiments with similar results.

Having observed that rejection of CaP cells in female mice was mediated by the hematopoietic system, we asked if transferring a female hematopoietic system into male mice would render them resistant to TRAMP-C2 tumor challenge. We generated bone marrow chimeras in which male hosts were transplanted with female T-cell depleted bone marrow. Despite having a hematopoietic system of female origin, TRAMP-C2 tumors grew readily in these chimeric mice (Figure 2A). This suggests that female-derived T cells are tolerized to prostate antigens when they develop in a male host. Tolerance of these cells may be both due to thymic “education” mediated by male radioresistant thymic epithelial cells, and by peripheral tolerance mechanisms engaged when these female T cells encounter the prostate and other male tissues.

To bypass central tolerance mechanisms, we next evaluated the potential of mature female lymphocytes to transfer anti-CaP immunity. These cells have undergone thymic selection in the female donor prior to transfer, and, therefore, should contain T cells capable of recognizing Y chromosome, androgen-regulated, prostate tissue-specific, and mutational antigens. We administered to male mice a nonmyeloablative dose of radiation to induce lymphopenia and create space for our transferred cells. Later the same day, mice were challenged with TRAMP-C2 tumor cells. One day later, either male or female splenocytes were infused into these tumor-challenged mice. We found that while infusion of male splenocytes had no effect on tumor growth, female splenocytes mediated a significant delay in tumor growth and improved tumor-free survival, in some cases leading to complete tumor rejection (Figure 2B).

By transferring total splenocytes, we are transferring CD4+CD25+FoxP3+ regulatory T cells (Treg) in addition to the effector T cells responsible for the GVT effects. It is possible, particularly using male donor mice, that these Treg cells may impede the efficacy of the transferred effectors. To ensure that our observations were not being compromised by the presence of Treg cells, we used magnetic beads to deplete Tregs from the adoptively transferred lymphocytes leaving less than 1% FoxP3+ cells remaining in the graft (data not shown). Depletion of Treg cells did not improve the efficacy of either female or male adoptively transferred lymphocytes (Figure 2C). This suggests that the primary determinant of GVT efficacy is the antigen specificity of the transferred effector cells, and that donor Treg cells do not have a significant impact on GVT.

To clarify the relative contributions of CD4 and CD8 T cells in mediating control of tumor growth by female splenocytes, we performed a mix-and-match experiment using male and female donors that were depleted *in vivo* with antibodies against either CD4 or CD8. Interestingly, we found that transfer of female CD4 T cells mediated similar tumor control when transferred with CD8 T cells of either male or female origin, while male CD4 T cells were unable to mediate tumor control even when infused simultaneously with female CD8 T cells (Figure 2D). Altogether, these results suggest that naïve CD4 T cells from female mice are essential for controlling growth of TRAMP-C2 tumors in male mice, while female CD8 T cells are not required.

### Female DBY-specific CD4 T Cells Potentiate the CD8 T-cell Response Against TRAMP-C2 Tumor Antigens

To confirm that CD4 T cells play a pivotal role in directing female immune responses against prostate cancer, we performed experiments to identify T cell responses against specific epitopes of antigens expressed by TRAMP-C2. Previously, we identified SPAS-1, an antigen on chromosome 2 expressed by TRAMP-C2 with an immunodominant class I epitope arising from a spontaneous point mutation [17]. By RT-PCR, we also identified expression of known class I-restricted Y chromosome antigens including SMCY, UTY, and EIAFIAY, as well as expression of class II-restricted Y chromosome antigen DBY (data not shown). It has been reported Y antigens are often selectively overexpressed in human CaP cell lines and tumors [7]. Similarly, we found that the genes for *smcy*, *uty*, and *dby* appeared to be selectively overexpressed in the TRAMP-C2 cell line and in spontaneously arising prostate tumor from TRAMP mice compared to normal B6 prostate (Figure S1).

**Figure 3:**
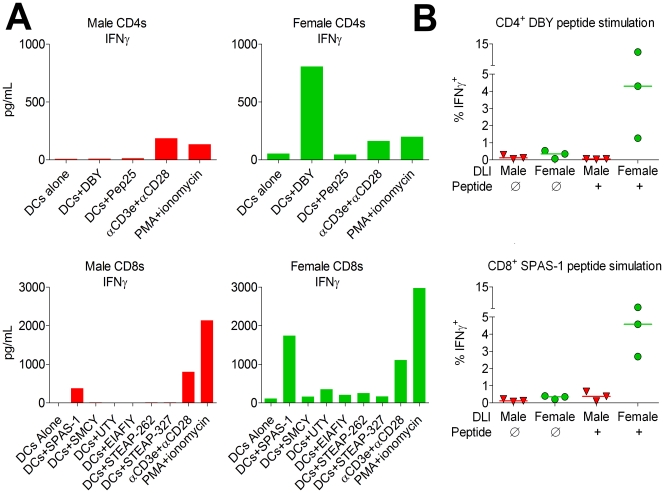
Female lymphocytes strongly recognize the CD4 antigen DBY and the CD8 antigen SPAS-1. A) Immunization of females with TRAMP-GMCSF cells produces augmented CD4 responses against DBY and CD8 responses against SPAS-1. Male and female B6 mice were injected intradermally with 1×10^6^ irradiated TRAMP-GMCSF at days −9, −6, and −3 prior to sacrifice. CD4+ and CD8+ splenocytes were purified with magnetic beads and restimulated with CD11c+ female splenic DCs pulsed with the indicated peptide, or alternatively were stimulated with anti-CD3ε/anti-CD28 beads, or with phorbol myristate acetate (PMA) and ionomycin. Supernatant concentrations of IFN-γ and other cytokines were assayed by BD cytometric bead array. Results of a single experiment. B) DNA vaccination with SPAS-1 produces immune responses in male mice adoptively transferred with female lymphocytes. Male B6 mice were given 6 Gy irradiation followed by male or female adoptive lymphocyte transfer. Mice were vaccinated by gene gun with SPAS-1-expressing DNA plasmids on days 1, 7, and 13 following adoptive transfer. On day 19, splenocytes were incubated with or without the SPAS-1 and DBY peptides, and production of IFN-γ by CD4 and CD8 T cells was evaluated by intracellular flow cytometry. Results of a single experiment.

To compare immune responses to TRAMP-C2 antigens arising in male and female mice, we utilized a cellular vaccine composed of irradiated GM-CSF-overexpressing TRAMP-C2 cells (TRAMP-GM-CSF) which we had previously generated and characterized [19]. We assayed vaccinated male and female mice for CD4 and CD8 T cells responses against known and predicted peptide epitopes derived from these antigens. Following re-stimulation with peptide-pulsed dendritic cells, we evaluated T-cell production of IFN-γ. We found that vaccination of female mice with TRAMP-GM-CSF results in strong CD4 T cell responses against DBY, while male mice do not develop measurable responses against DBY, though female and male CD4 T cells had similar responses to stimulation with anti-CD3ε/anti-CD28 beads, or with phorbol myristate acetate (PMA) and ionomycin (Figure 3A). Likely this is due to the lack of central tolerance against DBY in female versus male mice and the high expression of DBY by TRAMP-C2 cells. We found that male mice do mount CD8 T cell responses against the mutated antigen SPAS-1 following TRAMP-C2 cell vaccination. Female CD8 T cells, however, exhibit stronger responses to SPAS-1 than males (Figure 3A), an unexpected finding given that expression of SPAS-1 is neither gender-restricted nor hormone-regulated. The responses to SPAS-1 was dominant over responses to other antigens, including Y-chromosome antigens SMCY, UTY, and EIAFIY, as well as responses to the six transmembrane epithelial antigen of the prostate (STEAP), which had been reported due to be immunogenic in male mice and selectively overexpressed in prostate tumors [20]. Altogether, these results suggest that female DBY-specific CD4 T cells are able to potentiate CD8 T cell responses against an immunodominant tumor antigen arising from a spontaneous mutation.

To determine if a similar phenomenon of DBY-specific CD4 T cell “help” was occurring in the setting of adoptive cellular therapy, we transferred either male or female splenocytes into male hosts and then vaccinated with a DNA plasmid expressing mutated SPAS-1. We found that male mice that received female splenocytes spontaneously developed CD4 T-cell responses against DBY, evidenced by intracellular production of IFN-γ. Remarkably, males that received female splenocytes developed strong CD8 T-cell responses to SPAS-1 vaccination, while males that received male splenocytes had no significant response to SPAS-1 vaccination (Figure 3B). Results of these adoptive transfer experiments indicate that possible mechanisms for improved control of tumor growth in male recipients of female lymphocytes include both CD4 and CD8 T-cell recognition of antigens expressed by TRAMP-C2.

### Adoptive Transfer of Female Lymphocytes into Lymphopenic Male Hosts does not Result in Significant GVHD

**Figure 4:**
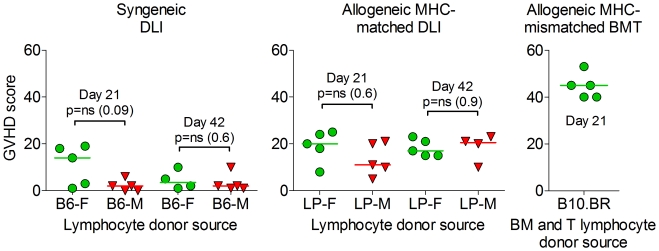
Adoptively transferred female lymphocytes do not produce increased graft-versus-host disease. Male B6 mice were given 6 Gy irradiation followed by an *i.v.* infusion of 3×10^7^ splenocytes, either male or female, from syngeneic B6 or allogeneic LP donors. For comparison, female B6 mice were lethally irradiated (11 Gy) and transplanted with 5×10^6^ T-cell depleted bone marrow cells and 2×10^6^ purified splenic T cells from MHC-mismatched B10.BR mice (H2k into H2b). Mice were evaluated on the day indicated for histological evidence of GVHD in the liver, small intestine, and large intestine. Results of a single experiment.

In our female-into-male immune transplant models, we have actively monitored for clinical signs of GVHD, which can include weight loss as well as changes in activity, posture, skin and fur. We have seen no evidence for GVHD in these experiments. In the bone marrow transplant setting, however, selecting a female donor can lead to an increased risk of GVHD [10]–[12]. In order to further investigate the safety of adoptive female cellular therapy for patients with prostate cancer, we evaluated histologically for subclinical evidence of GVHD in two models, B6 into B6 (syngeneic) and LP into B6 (MHC-matched, minor antigen-mismatched allogeneic), looking at GVHD target organs including liver, small intestine, and large intestine. We found that in the syngeneic setting, at two different time points (days 21 and 42 after transfer) female lymphocytes did not produce significantly more GVHD than male lymphocytes (Figure 4). In the more clinically relevant setting of an MHC-matched, minor-antigen mismatched model, we found a similar degree of GVHD using female and male donors at both early and late time points. Overall, GVHD pathological scores observed in these sublethal irradiation lymphocyte transfer models were mild in comparison to the MHC-mismatched bone marrow transplant with allogeneic T cell setting (Figure 4), confirming our observations that GVHD is mild in these models, and that the use of female lymphocytes does not appear to increase the incidence or severity of GVHD.

## Discussion

Here we report that adoptively transferred naïve female lymphocytes slow the growth of and, in some cases, reject pre-implanted TRAMP-C2 prostatic adenocarcinoma cells in sublethally irradiated male mice. A similar finding has also been recently reported by Yi et. al, who also used TRAMP-C2 cells [21]. They identified T cells as mediators of anti-prostate cancer immune responses in female mice and found that adoptive transfer of vaccinated female lymphocytes can lead to control of growth of TRAMP-C2 tumors in male hosts. Our study supports their initial findings and expands on them considerably by identifying mechanisms by which the female immune system mounts superior responses to prostate cancer. We demonstrate that female CD4 T cells are essential mediators of this effect, particularly those specific for the immunodominant epitope from the male antigen DBY. While Yi et. al found that female mice immunized with TRAMP-C2 developed some T-cell responses against normal prostate tissue as well as the prostate antigen STEAP, our data demonstrate that responses to STEAP are relatively minor in comparison with responses to the spontaneously arising immunodominant tumor antigen mutated SPAS-1, which was not assayed for in the previous study. Our results indicate that CD4 T cell responses against DBY, which are unique to female immune repertoires, can markedly augment CD8 T cell responses against a spontaneously arising mutant tumor antigen such as SPAS-1. CD4 T cell responses to DBY appear to be particularly robust in the setting of I-Ab MHC II restriction; T cells derived from mice bearing a transgenic T-cell receptor specific for the same DBY epitope have been previously shown capable of mounting responses against TRAMP-C2 tumors in RAG knockout mice [22]. We found that female lymphocytes in a male host were also able to mount superior responses to vaccination against SPAS-1, in the setting of concurrently developing spontaneous responses to DBY.

It is possible that CD4 T cells specific for DBY can supply help for CD8 T cells that mediate GVHD as well as GVT. However, by utilizing a model with sublethal irradiation followed by lymphocyte transfer without co-transfer of hematopoietic stem cells, we were able to markedly minimize graft-versus-host disease effects. Male mice treated with large doses of female lymphocytes had no clinical evidence for graft-versus-host disease and only very mild pathological evidence. At the same time, however, this approach may have allowed the establishment of tolerance to male and prostate antigens recognized by transferred female-derived T cells, potentially limiting the efficacy of this strategy and resulting in less mice fully rejecting their tumors.

Many of the recent advances in tumor immunotherapy have focused on either vaccination strategies or blockade of immune regulatory mechanisms. Two recent immune strategies that obtained FDA approval include vaccination against sipuleucel-T for metastatic CaP [1], and antibody-mediated blockade of the T-cell negative co-stimulatory receptor CTLA-4 for the treatment of metastatic melanoma [23]. The goal of vaccination strategies are to generate new anti-tumor T cell responses, while immune regulatory blockade strategies seek to release endogenous anti-tumor immune responses that are generated spontaneously but restrained and ineffective [24]. While both approaches show promise, clinical data show that most patients eventually still succumb to progression of their cancer. Combinations of immune approaches may be the most promising. In this study, we identify a third approach, namely enhancement of the immune repertoire by adoptively transferring sex-mismatched lymphocytes with improved ability to recognize tumor antigens. Repertoire enhancement has the potential to dynamically synergize with both tumor vaccination and immune regulatory blockade strategies.

For metastatic castration-resistant prostate cancer, a disease which continues to have a poor prognosis, we show that female lymphocytes, particularly CD4 T cells, adoptively transferred into prostate-cancer challenged male mice can mediate substantial anti-tumor effects. In terms of safety, this approach appears clinically viable as we observed no substantial GVHD in either syngeneic or MHC-matched, minor mismatched allogeneic female to male lymphocyte transfers. The data presented here supports further evaluation of this approach to prostate cancer immunotherapy and its potential to synergize with other supportive immunotherapeutic approaches.

## Materials and Methods

### Mice

All mouse procedures were performed in accordance with institutional protocol guidelines at Memorial Sloan-Kettering Cancr Center (MSKCC). Mice were maintained according to NIH Animal Care guidelines, under protocols approved by the MSKCC Institutional Animal Care Committee describing experiments specific to this study. C57BL/6 and LP mice were obtained from the Jackson Laboratory.

### Antibodies

CD4 (GK1.5) and CD8 (2.43) depleting antibodies were purchased from Bio-Xcell. Flow cytometry was performed using antibodies recognizing CD45 (30-F11), CD4 (RM4–5), CD8α (53–6.7), IFN-γ (XMG1.2) purchased from BD Biosciences.

### Peptides

All peptides used were purchased from Biosynthesis, Inc. and used at a final concentration of 10 µm. CD8 peptides included SPAS-1 (244–252), SMCY (738–746), UTY (246–254), EIAFIY (63–71) and STEAP (262–270, 327–335). CD4 peptides included DBY (608–622) and as a control, Pep25, the dominant MHC class II-presented epitope of M. *tuberculosis* Ag85B [25].

### Cell Lines

TRAMP-C2 tumor cells were provided by Dr. Norman Greenberg [26] and cultured as previously described [16]. TRAMP-GM-CSF cells have also been previously described [19].

### BMT

All transplantation protocols were reviewed and approved by the Memorial Sloan-Kettering Cancer Center IACUC. Transplantation protocols have been previously described [27].

### Surgical Castration

Mice were anesthetized and a small scrotal incision made to reveal the testes. These were sutured and removed along with surrounding fatty tissue. The wound was closed using surgical staples.

### TRAMP-C2 Gender Challenge

5-week old male, 8 week old male, and 8 week old female mice were challenged with 5×10^5^ TRAMP-C2 cells intradermally on the right flank and monitored for tumor growth.

### Lymphocyte Adoptive Transfer

Recipient mice were rendered lymphopenic with non-myeloablative (6 Gy) whole body irradiation. Following irradiation, mice were rested for 4–8 hours and then implanted with 1×10^6^ TRAMP-C2 cells intradermally. On the following day, splenocytes were purified from 8–10 week old donor male or female C57BL/6 mice and 3×10^7^ per mouse were transferred via tail vein injection to the recipient animals. Treg cells were removed from transferred lymphocyte populations where indicated using the Miltenyi Biotec CD4+CD25+ isolation kit according to the manufacturer’s instructions. For CD4 and CD8 mix and match experiments, mice were either depleted of CD4 T cells with the GK1.5 antibody (for CD8 transfer) or depleted of CD8 T cells with the 2.43 antibody (for CD4 transfer). Depletions consisted of 2 injections of 500ug of the depleting antibody at days −6 and −2 prior to transfer. Mix and match adoptive transfers were performed as above except that 1.5×10^7^ splenocytes from various CD4 depleted mice were transferred in combination with 1.5×10^7^ splenocytes from the indicated CD8 depleted mice.

### SPAS-1 DNA Vaccination

On days 1, 7, and 13 following irradiation and lymphocyte transfer, mice were vaccinated by gene gun with SPAS-1-expressing DNA plasmids as previously reported [28]. Briefly, plasmid DNA was purified, coated onto 1-µm diameter gold particles (Alfa Aesar, Ward Hill, MA), and allowed to settle on bullets of Teflon tubing. Gold particles containing 1 µg DNA were delivered to each abdominal quadrant by the use of a helium-driven gene gun (Accell; PowderMed, Oxford, United Kingdom), for a total of 4 µg DNA per mouse. Mice were vaccinated on days 1, 7, and 13 following DLI. On day 19, splenocytes were incubated with or without the SPAS-1 and DBY peptides in the presence of GolgiPlug (BD Biosciences), and production of IFN-γ by CD8 and CD4 T cells was evaluated by intracellular flow cytometry.

### TRAMP-specific T-cell Responses

Male and female C57BL/6 mice were vaccinated with 1×10^6^ TRAMP-C2 cells expressing GM-CSF intradermally at days −9, −6, and −3 prior to sacrifice. Draining lymph nodes were removed and CD4 and CD8 T cells were purified using positive selection beads from Miltenyi Biotech. Female CD11c+ dendritic cells were purified from spleens of naïve mice using Miltenyi positive selection beads and pulsed with 10 µm of the indicated peptide for 6–8 hours. Purified T cells were incubated with peptide pulsed DCs and production of cytokines including IFN-γ was measured after 48 hours of incubation using the BD TH1/TH2/TH17 cytometric bead array as previously described [29]. Positive controls included purified T cells from vaccinated mice polyclonally activated using either Mouse T-activator αCD3/CD28 beads (Life Technologies) or using 50 ng/ml phorbol myristate acetate (PMA) and 1 µg/ml ionomycin (Sigma).

### Evaluation of GVHD

Mice were monitored daily for survival and weekly for GVHD clinical scores [30]. Small intestine, large intestine, and liver samples were evaluated histologically for evidence of GVHD and scored as previously described [31].

## Supporting Information

Figure S1 **Selective overexpression of some Y-antigens by TRAMP tumors.** RNA was purified from the indicated tumor cell line or primary tissue using TRI-ZOL (Invitrogen). The RNA was reverse-transcribed into cDNA using the Superscript II RT Kit (Invitrogen). PCR was then performed using Taq Polymerase Mastermix from Qiagen and gene-specific primers as indicated. β-Actin was amplified in parallel to standardize for cDNA content and quality between samples.
(TIF)
