# Supplementary figures and images for: Repertoire Enhancement with Adoptively Transferred Female Lymphocytes Controls the Growth of Pre-Implanted Murine Prostate Cancer
Source: PLoS One. 2012 Apr 6;7(4):e35222. doi: 10.1371/journal.pone.0035222 (PMC3320876; doi:10.1371/journal.pone.0035222)

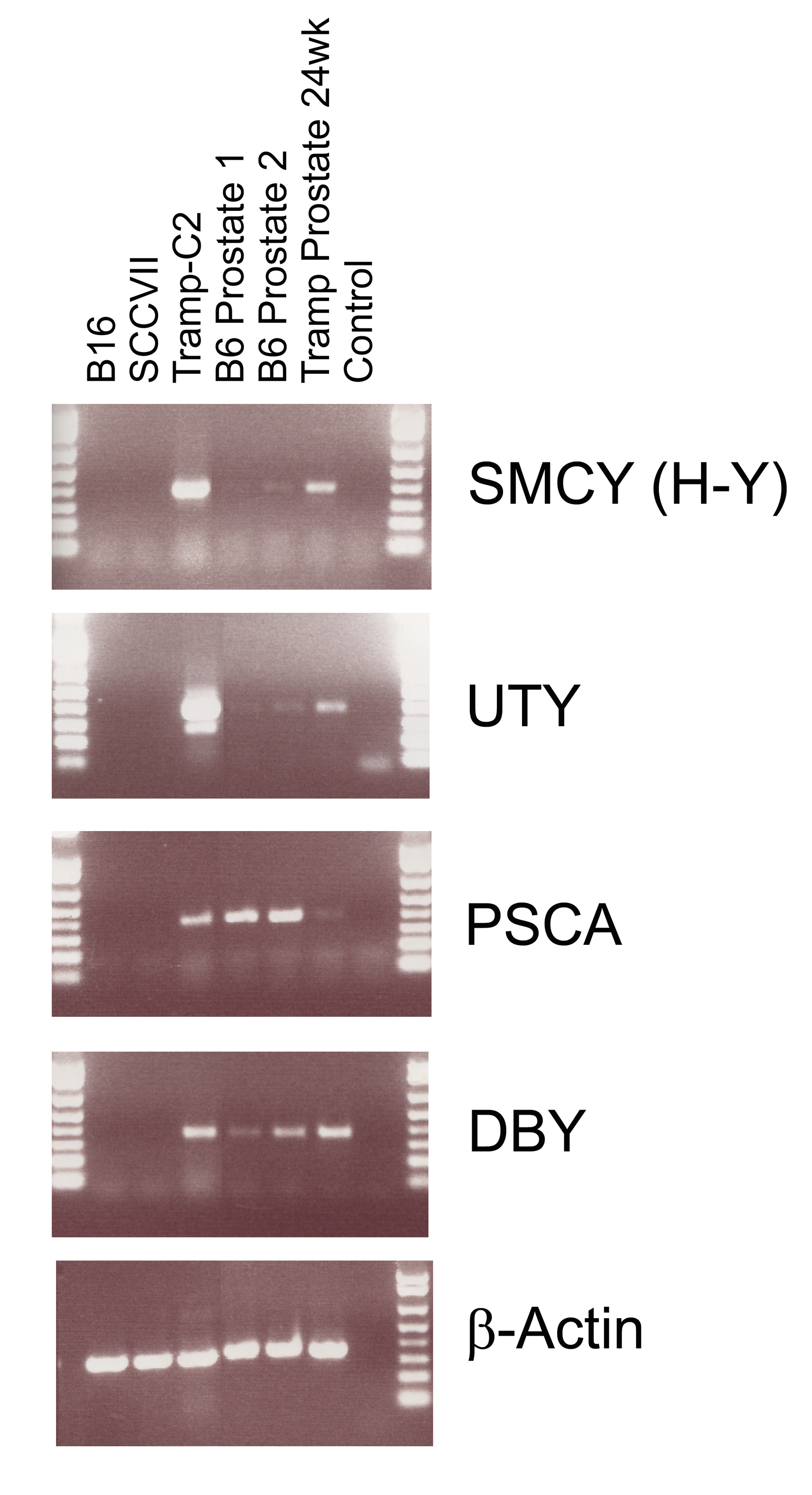

Supplement: Figure S1 — Selective overexpression of some Y-antigens by TRAMP tumors. RNA was purified from the indicated tumor cell line or primary tissue using TRI-ZOL (Invitrogen). The RNA was reverse-transcribed into cDNA using the Superscript II RT Kit (Invitrogen). PCR was then performed using Taq Polymerase Mastermix from Qiagen and gene-specific primers as indicated. β-Actin was amplified in parallel to standardize for cDNA content and quality between samples. (TIF) [file pone.0035222.s001.tif]
